# Accurately Predicting Glutarylation Sites Using Sequential Bi-Peptide-Based Evolutionary Features

**DOI:** 10.3390/genes11091023

**Published:** 2020-08-31

**Authors:** Md. Easin Arafat, Md. Wakil Ahmad, S.M. Shovan, Abdollah Dehzangi, Shubhashis Roy Dipta, Md. Al Mehedi Hasan, Ghazaleh Taherzadeh, Swakkhar Shatabda, Alok Sharma

**Affiliations:** 1 Department of Computer Science and Engineering, United International University, Dhaka 1212, Bangladesh; marafat152047@bscse.uiu.ac.bd (M.E.A.); mahmad152213@bscse.uiu.ac.bd (M.W.A.); iamdipta@gmail.com (S.R.D.); 2 Department of Computer Science and Engineering, Rajshahi University of Engineering and Technology, Rajshahi 6204, Bangladesh; sm.shovan@gmail.com (S.M.S.); mehedi_ru@yahoo.com (M.A.M.H.); 3 Department of Computer Science, Rutgers University, Camden, NJ 08102, USA; i.dehzangi@rutgers.edu; 4 Center for Computational and Integrative Biology, Rutgers University, Camden, NJ 08102, USA; 5 Institute for Bioscience and Biotechnology Research, University of Maryland, College Park, MD 20742, USA; 6 Institute for Integrated and Intelligent Systems, Griffith University, Brisbane, QLD 4111, Australia; 7 Department of Medical Science Mathematics, Tokyo Medical and Dental University (TMDU), Tokyo 113-8510, Japan; 8 Laboratory for Medical Science Mathematics, RIKEN Center for Integrative Medical Sciences, Yokohama, Kanagawa 230-0045, Japan; 9 School of Engineering and Physics, Faculty of Science Technology and Environment, University of the South Pacific, Suva, Fiji

**Keywords:** post-translational modification, lysine Glutarylation, machine learning, extra-trees classifier, bi-peptide evolutionary features

## Abstract

Post Translational Modification (PTM) is defined as the alteration of protein sequence upon interaction with different macromolecules after the translation process. Glutarylation is considered one of the most important PTMs, which is associated with a wide range of cellular functioning, including metabolism, translation, and specified separate subcellular localizations. During the past few years, a wide range of computational approaches has been proposed to predict Glutarylation sites. However, despite all the efforts that have been made so far, the prediction performance of the Glutarylation sites has remained limited. One of the main challenges to tackle this problem is to extract features with significant discriminatory information. To address this issue, we propose a new machine learning method called BiPepGlut using the concept of a bi-peptide-based evolutionary method for feature extraction. To build this model, we also use the Extra-Trees (ET) classifier for the classification purpose, which, to the best of our knowledge, has never been used for this task. Our results demonstrate BiPepGlut is able to significantly outperform previously proposed models to tackle this problem. BiPepGlut achieves 92.0%, 84.8%, 95.6%, 0.82, and 0.88 in accuracy, sensitivity, specificity, Matthew’s Correlation Coefficient, and F1-score, respectively. BiPepGlut is implemented as a publicly available online predictor.

## 1. Introduction

Post-translational modifications (PTMs) of proteins are associated with various biological processes. They also play a vital role in the diversification of protein functioning in different biological and physiological interactions [1,2]. PTMs are associated with different functions such as systematizing biological activities and regulating localization, and proteins interacting with other cellular molecules. PTMs are also key components of biological processes for the transmission of the genetic code and the control of cellular physiology. In 2016, Trost et al. [3] described the DAPPLE2 tool to predict 20 different types of PTMs from 15 online databases. DAPPLE2 is able to make the prediction task faster than its previous version, DAPPLE [4]. Later on, Li et al. [5] developed a new R package, named PTMscape, that predicts PTM sites based on diverse sets of physicochemical-modified properties. More recently, Chen et al. [6] introduced MUscADEL tools for the PTMs prediction using deep learning. So far, more than 600 types of PTMs have been identified. Some of the most widely observed PTMs are Acetylation [7], Propionylation [8], Sumoylation [9], Succinylation [10,11], Malonylation [12], and Methylation [13,14] among the main 20 contributing amino acids to build proteins [15].

Lysine Glutarylation is among the recently identified PTMs. Glutarylation occurs when an amino acid along the protein sequence interacts with a glutaryl group. Glutarylated proteins have been identified for many metabolic procedures and mitochondrial functions in both eukaryotic and prokaryotic cells [16]. Among the most important ones, Glutarylation dysregulation has been related in the etiology of metabolic disorders such as cancer [17], mycobacterium tuberculosis [18], diabetes, and brain and liver disorders [19]. Therefore, due to the tangled characteristic and limited knowledge of Glutarylation sites, further analysis for a better understanding of the nature of Glutarylation is required.

During the past few years, a wide range of methods has been proposed to predict Glutarylation sites using many machine learning approaches [20,21,22,23,24,25]. Recently, many deep learning models have been used to predict different types of PTMs [6,26,27,28,29]. In one of the earliest studies, Tan et al. detected 23 Glutarylation sites in 13 unique proteins from HeLa cells [16]. They also examined 683 lysine Glutarylation sites in 191 individual proteins. After that, Xie et al. also identifies 41 Glutarylation sites in 24 Glutarylated proteins. They extracted features based on the composition of amino acids and amino acid interactions and used Support Vector Machine (SVM) as their classifier [18].

In a different study, López et al. proposed structural features and evolutionary information of amino acids to predict the succinylation sites, which is closely related to Glutarylation sites prediction [20,21]. Recently, Zhe et al. [22] developed a predictor tool named GlutPred. To predict the Glutarylation sites, they extracted different kinds of features and applied a maximum relevance minimum redundancy feature selection method. They also used a biased SVM classifier to build GlutPred. At the same time, Yan et al. [23] proposed another predictor, called iGlu-Lys, to tackle this problem. They used a wide range of features and selected the optimal features using special-position information and amino acid pair order. They also used SVM as their preferred classifier. More recently, Huang et al. [24] proposed a new model called MDDGlutar. To build this model, they used sequence-based features such as Amino Acid Composition (AAC), Amino Acid Pair Composition (AAPC), and Composition of k-spaced Amino Acid Pairs (CKSAAP). They also employed the SVM classifier to identify the Glutarylation sites. Most recently, Hussam et al. [25] developed another tool, named RF-GlutarySite, that uses sequence-based and physicochemical-based features and employs Random Forest (RF) as a classifier.

Despite all the efforts that have been made so far, the overall performance of the lysine Glutarylation site prediction task remained limited. The main challenge to enhance lysine Glutarylation site prediction performance is the use of features that provide significant discriminatory information. In this paper, we propose a new model called BiPepGlut that uses a bi-peptide-based evolutionary feature extraction concept to enhance lysine Glutarylation prediction performance. We investigate the impact of several classifiers and choose the one with the best performance to build our model. Among them, Extra-Trees (ET) classifier outperforms other classifiers, which is used to build BiPepGlut.

**Figure 1:**
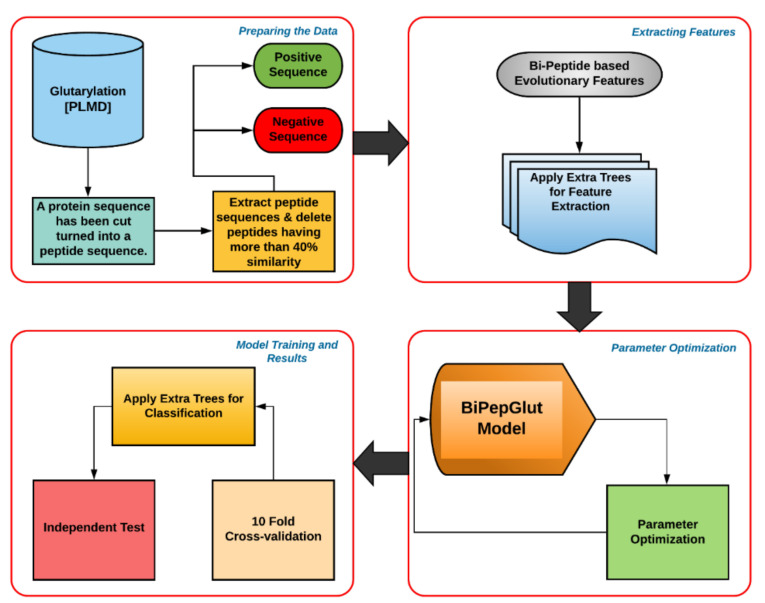
This flow chart demonstrates the general architecture of BiPepGlut. The positive and negative sites were yielded from a public database. Features were then extracted using the bi-peptide-based evolutionary feature extraction technique and then the useful features are selected. After that, the Extra Tree (ET) classifier was trained using our extracted features and then evaluated using 10-fold cross-validation and an independent test set.

The entire methodology is described in detail in the following sections. An overview of the general architecture of BiPepGlut is given in Figure 1. Our results demonstrate that BiPepGlut is able to significantly enhance lysine Glutarylation prediction accuracy compared to those methods found in the literature. BiPepGlut achieves 92.0%, 84.8%, 95.6%, 0.82, and 0.88 in accuracy, sensitivity, specificity, Matthew’s Correlation Coefficient (MCC), and F1-score on the employed independent test, respectively. Such results demonstrate more than 3% improvement for sensitivity, and over 0.3 improvements for MCC compared to those reported in the previous studies. BiPepGlut is implemented as an online predictor and is publicly available at: www.brl.uiu.ac.bd/bioglutarylation/. The data, code, and all the Supplementary Materials used to build the BiPepGlut method are publicly available at: https://github.com/Wakiloo7/BipepGlut.

## 2. Materials and Methods

In this section, we present our employed benchmark, how it is prepared for further experimentation, our employed classifiers, proposed feature extraction, and measurement methods.

### 2.1. Dataset

In this study, we collected a Glutarylation dataset from Protein Lysine Modifications Database (PLMD) [30]. The PLMD repository contains datasets for different PTM sites. All the PTMs recorded in this repository are those that are interacted with the lysine amino acid along the protein sequence. It is mainly because lysine has a high tendency to engage in PTM interaction compared to other amino acids. This dataset contains 211 proteins, which have 715 lysine Glutarylation sites belonging to *Mus musculus* (mouse) and *Mycobacterium tuberculosis* species. Among them, 674 sites in 187 proteins and 41 sites in 24 proteins belong to *Mus musculus* and *Mycobacterium tuberculosis*, respectively. We then cut the protein sequences into peptides by considering window size as 21. This window size has been widely used in the literature and shown to be the best among other window sizes [9,23,25].

**Figure 2:**
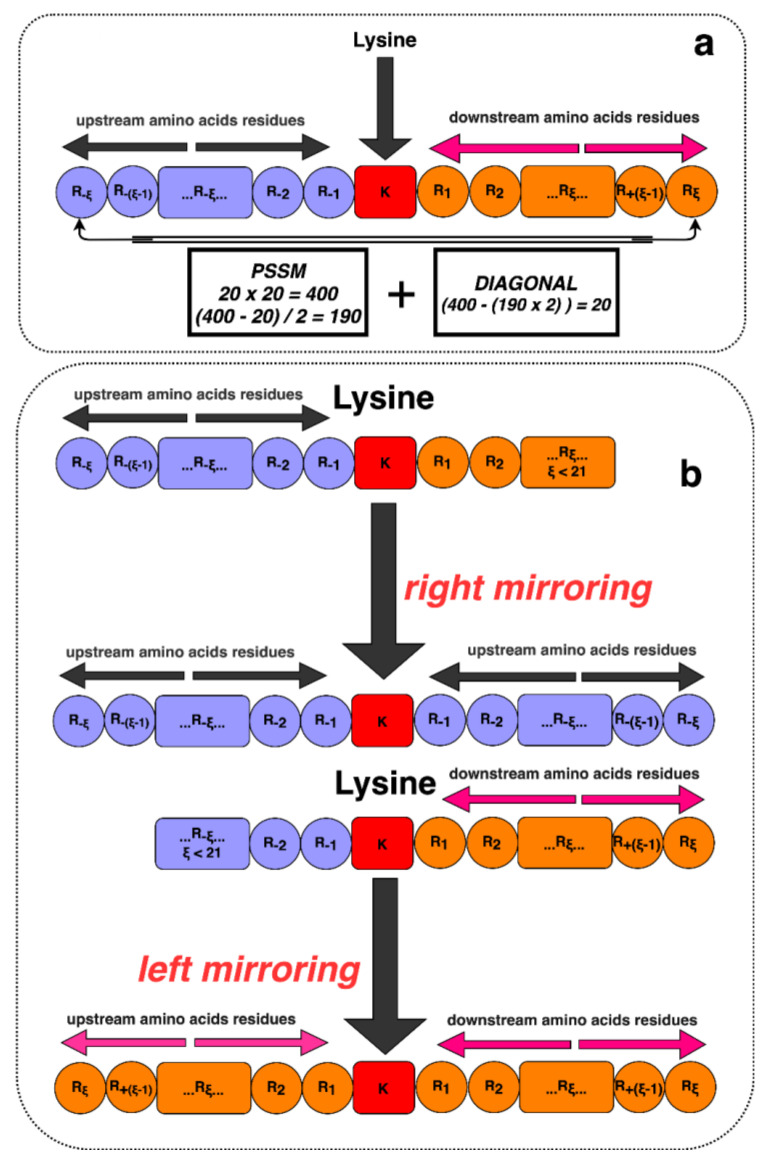
Illustration of lysine residues with its surrounding upstream and downstream amino acids. (**a**) Lysine residues with sufficient neighboring amino acids. (**b**) A scenario of adding dummy residues in N-terminus and C-terminus to have insufficient amino acids neighbors on either the upstream or downstream segment.

For better representation, we use an alphabet notation, where the upstream and downstream lengths are denoted as *ξ* = 10, and the entire window size is 2*ξ* + 1 (2 × 10 + 1 = 21). The responsible residue for the Glutarylation site is one letter notation of *K* (amino acid lysine). Alongside this, a dummy residue *(X)* has been added on both sides of the proteins when the lysine is in the N-terminus or C-terminus of the proteins and does not have 10 neighboring amino acids in both sides to ensure the uniform length upstream and downstream. This process is shown in Figure 2.

As a result, we have a total of 723 Glutarylation sites (positive) and 4626 Non-Glutarylation (negative) sites. Later on, we applied CD-HIT [31] over the negative sequences to remove sequences with high sequential similarity. In this case, we use 40% similarity cut-off as it is widely used in the literature [23,24,25]. Due to the limited availability of positive samples compared to the negative samples, the peptides with positive sites remain untouched. To provide more insight into our employed benchmark, we produce ranking of homology in the positive and negative hits separately using CD-HIT, which is now available at: https://github.com/Wakiloo7/BiPepGlut/tree/master/CD-HIT. In this way, we can avoid underfitting our model in predicting positive sites. However, we use both 10-fold cross-validation and an independent test set to investigate the generality of our model and to avoid bias in our model. As a result of using CD-HIT, the 1923 Non-Glutarylation sequence remains from the original 4626 negative sites. We cross-checked positive sequences in the negative sites to make sure about the validity of our employed benchmark. From the remaining samples, we randomly separate 90% of the samples to build the training set while the remaining 10% to build the independent test set.

### 2.2. Feature Extraction

Feature extraction is an important step in building an effective and accurate machine learning model. In general, feature extraction is the method of selecting, handling, and managing a set of *F* features from a given dataset. For our case, the employed data set contains protein sequences. A wide range of feature extraction techniques has been proposed in the literature to extract discriminatory information to represent protein sequences [20,21,32,33]. Most of the extracted features for Glutarylation site predictions are based on the physicochemical or alphabetic sequential properties of the proteins. However, the other sources for feature extraction such as evolutionary-based features have not been adequately explored for the Glutarylation site prediction task [34,35,36]. In this scenario, we focus on extracting evolutionary-based features using the bi-peptide method to tackle this problem.

### 2.3. Bi-Peptide-Based Evolutionary Feature Extraction Technique

Peptide is a molecule consisting of two or more amino acids. Peptides are usually shorter than proteins. Our proposed concept includes bi-peptide-based evolutionary feature extraction techniques to predict the Glutarylation sites. This technique has been effectively used for similar studies [33,34,35,36,37,38,39,40]. We extract the features straight from the Position Specific Scoring Matrix (PSSM) as one of the most important resources to extract evolutionary information. PSSM matrix is produced as the output of the Position-Specific Iterative Basic Local Alignment Search Tool (PSI-BLAST) [41]. PSI-BLAST aligns a given peptide sequence with a protein database to identify similar sequences and produces PSSMs. These PSSMs specify the substitution score of a given amino acid of a protein sequence compared with other protein sequences. Such a substitution score determines the possibility of a given amino acid is substituted to other amino acids due to evolutionary changes. In this case, we execute PSI-BLAST using three iterations and a cut-off e-value (*E*) 0.001 to generate the PSSM matrix.

In this study, Glutarylated (positive) and Non-Glutarylated (negative) sites and their neighboring amino acids (10 upstream and 10 downstream of amino acids) were allied to extract the features. In this scenario, these neighboring amino acids are presented with the *P_ξ_(K)* segment of sequences. For example, a peptide sample can be presented as:

*P_ξ_(K) = R*_−ξ_*R*_−(ξ−1)_…*R*_−2_*R*_−1_ ʘ *R*_1_*R*_2_…*R*_+(ξ−1)_*R*_+ξ_ (1)

The central amino acid expresses as lysine (K) is indexed as *ξ*. The downstream is indicated as *R_+ξ_* and the upstream is denoted as *R_−ξ_*. A substring of the protein sample is (2*ξ* + 1), which is the entire length of the peptide sequence. Two categories are shown in this case where each peptide samples fall under them.

(2) $$P_{\xi }\left(K\right)\in \left\{\begin{array}{ll} P_{\xi }^{+}\left(K\right), & \;if\;the\;responsible\;residue\;is\;a\;Glutarylation\;site\\ P_{\xi }^{1}\left(K\right), & \;otherwise \end{array}\right.$$

In this scenario, the negative Glutarylated set is denoted as *P_ξ_^−^(K)*, and the positive Glutarylated set is denoted as *P_ξ_^+^(K)*. As a result, we can introduce our benchmark dataset as:

*S*_ξ_(*K*) = *S*_ξ_^+^(*K*) ∪ *S*_ξ_^−^(*K*) (3)

where the Glutarylated set *P_ξ_^+^(K)* is presented in terms of *S_ξ_^+^(K)* and carries the Non-Glutarylated set *P_ξ_^−^(K)*, which is presented in terms of *S_ξ_^-^(K)* while ∪ describes the union operator. The following techniques are carried to produce the feature vector from our dataset.

(i) The peptide sequence can be presented by *P* that is constituted as:
  *P* = *R*_1_*R*_2_*R*_3_*R*_4_ … *R*_L_ (4)

From the study of Schaffer et al. [41], *P* can be demonstrated by an L×20 dimensional matrix, which is shown as:

(5) $$\left[\begin{array}{cccc} \overset{´}{E_{1\to 1}} & \overset{´}{E_{2\to 1}} & \ldots & \overset{´}{E_{L\to 1}}\\ \overset{´}{E_{1\to 2}} & \overset{´}{E_{2\to 2}} & \ldots & \overset{´}{E_{L\to 2}}\\ \vdots & \vdots & & \vdots \\ \overset{´}{E_{1\to 20}} & \overset{´}{E_{2\to 20}} & \ldots & \overset{´}{E_{L\to 20}} \end{array}\right]$$

Here, *L* refers to the length of *P*, and *É_i__→__j_* refers to 20 different amino acids that get propensity of the amino acid residue spread.

(ii) From Equation (5), we generate the transpose matrix as:
  (6) $$\left[\begin{array}{cccc} E_{1\to 1} & E_{2\to 1} & \ldots & E_{L\to 1}\\ E_{1\to 2} & E_{2\to 2} & \ldots & E_{L\to 2}\\ \vdots & \vdots & & \vdots \\ E_{1\to 20} & E_{2\to 20} & \ldots & E_{L\to 20} \end{array}\right]$$
  with,
  (7) $$E_{i\to j}=\frac{\overset{´}{E_{i\to j}}-\;\bar{\overset{´}{E_{j}}}}{SD\left(\bar{\overset{´}{E_{j}}}\right)}\;i=1,\;2,\;3,\;\ldots ,\;L;\;j=1,\;2,\;\ldots ,\;20$$
  where,
  (8) $$\bar{\overset{´}{E_{j}}}=\;\frac{1}{L}\;\overset{L}{\underset{i=1}{\sum }}\overset{´}{E_{i\to j}}\;\;j=1,\;2,\;\ldots ,\;20$$

The standard deviation is calculated and denoted where *É* denotes the mean of *É_i__→__j_* for *i* = 1, 2, …, 20 by the following equation.

(9) $$SD\left(\bar{\overset{´}{E_{j}}}\right)=\;\sqrt{\overset{L}{\underset{i=1}{\sum }}{\left[\overset{´}{E_{i\to j}}-\overset{´}{E_{j}}\right]}^{2}/\;L}$$

(iii) The newly created matrix *M^T^M* is evaluated by the matrix *M* as well as the transpose of a matrix, which, in turn, is a 20 × 20 matrix (20×*L* × *L*×20 = 20 × 20 matrix) of 400 elements. The transpose matrix *M^T^* multiplies with the main *M* matrix. The resulting matrix is symmetric. Therefore, the upper or lower triangular plus the main diagonal will have all the information that is extracted in this matrix. The triangular matrix inhibits 210 features where the first 20 comes from the diagonal and the rest 190 ((400 − 20)/2) features are from either lower or upper triangle matrices (190 + 20 = 210 total), as shown below.
  (10) $$\left[\begin{array}{ccccc} \left(1\right) & & & & \\ \left(2\right) & \left(3\right) & & & \\ \left(4\right) & \left(5\right) & \left(6\right) & & \\ \vdots & \vdots & \vdots & & \\ \left(191\right) & \left(192\right) & \left(193\right) & \ldots & \left(210\right) \end{array}\right]$$

The new matrix was then converted to a vector consisting of 210 elements, which can be represented as follows.

(11) $$P_{evo}=\left[\Theta_{1}^{E},\;\Theta_{2}^{E},\;\ldots ,\;\Theta_{u}^{E},\ldots ,\Theta_{210}^{E}\right]$$

### 2.4. Handling Imbalanced Dataset

As explained in the Dataset Subsection, the number of Glutarylation sites (positive) is lesser than the number of Non-Glutarylation sites (negative). There are significantly more negative samples in our benchmark when compared to positive samples. Such an imbalance may lead the predictor to be biased toward the negative samples. To avoid such a bias, it is necessary to balance the employed dataset. To deal with this issue, various balancing schemes have been introduced in the literature [42,43,44].

To address this issue, we up-sample positive sites (Glutarylation) instead of down-sampling the negative sites (Non-Glutarylation). Down-sampling may reduce the important usable samples. In this study, we use an oversampling approach by creating well-characterized synthetic data [45,46,47]. To ensure the little variation based on the property of the dataset, we pick the maximum value of the entire feature vectors. We then multiply the positive sites to 1.0001 and 1.0005, where the new value is much closer to the original value as done in References [13,38]. Initially, we have 723 positive sites. Multiplying 723 positive sites with 1.0001 and 1.0005 (723 + (723 × 1.0001) + (723 × 1.0005) = 2169), the new values are much closer to the original values. We generate our newly created value in this approach. Therefore, the number of positive sites increases to 2169, while the number of negative sites is 1923, where the ratio between positive and negative is almost ≈1. The overall balancing process is only applied to training data while the test data remain untouched. This is how we make sure to avoid over-fitting. Hence, the balancing strategy also contributes to diminishing bias.

### 2.5. Classification Techniques

Choosing the most useful classification technique is an essential step in building a machine learning method. In this study, we have applied different kinds of classification methods. These classifiers are also widely used in the literature and demonstrated promising results for similar studies [13,39,48,49,50]. In this case, we study several ensemble learning methods such as Extreme Gradient Boosting (XGBoost) [48], Extra Tree (ET) Classifier [49], and Random Forest (RF) [25]. We also investigate several meta-classifiers such as Adaptive Boosting (AdaBoost) [39] and a tree-based learning algorithm Light Gradient Boosting Machine (LightGBM) [13]. We also study several of the most popular classifiers such as the Multi-layer Perceptron (MLP) classifier, which is a popular Artificial Neural Network (ANN) model [50].

In this study, the implementation of these classifiers is from the Scikit-learn version 0.19.2. To implement these algorithms through the classification model, we have used the following hyperparameters. Among them, some are default parameters and the rest of the parameters are tuned as required. In XGBoost, we tuned n_estimators = 300. For ET classifiers, we used n_estimator = 10, min_sample_split = 2. For RF classifiers, max_depth = 2, random_state = 42, and n_estimators = 300. For AdaBoost, we use n_estimators = 300 while, for LightGBM, num_leaves = 31, learning_rate = 40, and n_estimators = 40. Lastly, for MLP, we use one hidden layer and 100 nodes, an activation function as Relu, α = 1, max_iter = 1000, and learning_rate_int = 0.001. During the hyperparameters tuning, among all the classifiers, we identify that the ET classifier attains the best results compared to other classifiers. Extra Trees (ET) classifier uses an ensemble learning method, which is a type of meta estimator that fits many decision trees similar to the RF classifier. In ET, selected features have been chosen randomly by splitting. In many cases, ET improves predictive accuracy and diminishes the chances of over-fitting [49,51].

### 2.6. Performance Measurements

In this case, we use both 10-fold cross-validation, and an independent test set to study the performance and generality of our proposed model. We also use accuracy, sensitivity, specificity, MCC, and F1-score as our performance measurements, which are used in previous studies [52,53]. Using these measurements, we will be able to directly compare our results with those reported in the earlier studies. These measurements are formulated as follows.

(12) $$Accuracy\;\left(ACC\right)=\left(1-\frac{GS_{-}^{+}+GS_{+}^{-}}{GS^{+}+GS^{-}}\right)\times 100$$

(13) $$Sensitivity\;\left(SN\right)=\left(1-\frac{GS_{-}^{+}}{GS^{+}}\right)\times 100$$

(14) $$Specificity\;\left(SP\right)=\left(1-\frac{GS_{+}^{-}}{GS^{-}}\right)\times 100$$

(15) $$MCC=1-\frac{\left(\frac{G_{-}^{+}}{GS^{+}}+\frac{G_{+}^{-}}{GS^{-}}\right)}{\sqrt{\left(1+\frac{G_{+}^{-}-\;G_{-}^{+}}{GS^{+}}\right)\left(1+\frac{G_{-}^{+}-\;G_{+}^{-}}{GS^{-}}\right)}}$$

(16) $$F1-score=2\times \frac{\left(PR\;\times RE\right)}{\left(PR+RE\right)}$$

where *GS^+^* denotes positive (Glutarylation) sites that are correctly classified, *GS*^−^ denotes negative (Non-Glutarylation) sites that are classified correctly, $GS_{-}^{+}$ indicates Non-Glutarylated peptides that are wrongly classified as Glutarylated, and $GS_{+}^{-}$, shows the Glutarylated peptides that are incorrectly predicted as Non-Glutarylated. Precision (PR) and Recall (RE) are also examined for the performance analysis along with the F1-score.

## 3. Results and Discussion

In this section, we will first present how we choose our employed classifier among a wide range of classifiers that we studied in this case. We then compare our results with the state-of-the-art models found in the literature and demonstrate the effectiveness of BiPepGlut. We then analyze our results.

### 3.1. Building Our Model by Choosing the Most Effective Classifier

In this case, we investigate and compare the performance of six machine learning algorithms: LightGBM [13], RF [25], AdaBoost [39], XGBoost [48], ET classifier [49], and MLP classifiers [50]. The results achieved for this comparison for the 10-fold cross-validation and independent test set are shown in Table 1 and Table 2, respectively, where ACC is accuracy, SN is sensitivity, and SP is specificity. As shown in these tables, among these classifiers, LightGBM and ET obtain the best results. Among these two, ET achieves relatively better results. 

**Figure 3:**
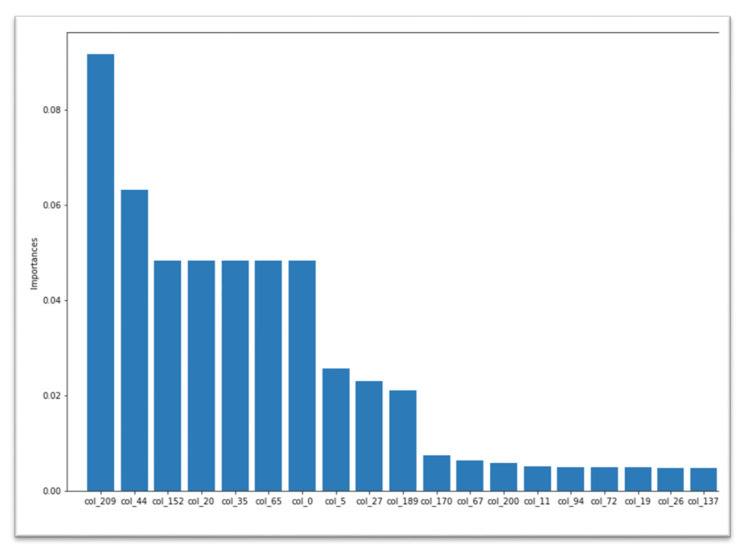
Feature importance of 210 features selected for our model development.

BiPepGlut also applies an Extra Trees (ET) classifier by using their Gini importance for computing the importance of features. To do this, we took each feature Gini importance and selected the top-most significant feature, according to their preference. The feature importance chart for our 210 lengths of features is shown in Figure 3. Note that, during model development, we exclusively work with these 210 features instead of skipping any features. Corresponding to all the results, from Table 1 and Table 2, the ET [49] classifier obtains better performance compared to other classifiers. It achieves 81.5% in accuracy, 70.0% in sensitivity, 92.9% in specificity, 0.64 in MCC, and 0.79 in F1-score. In addition, the true positive (TP) rates are 1322, 460, and the false positive (FP) rates are 101-fold, 40-fold, and 10-fold cross validation and an independent test set, respectively.

**Figure 4:**
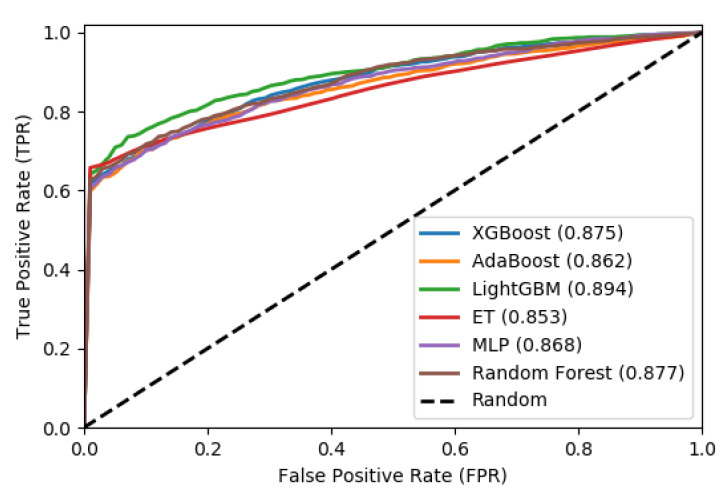
Receiver operator characteristic (ROC) curves using 10-fold cross-validation.

**Figure 5:**
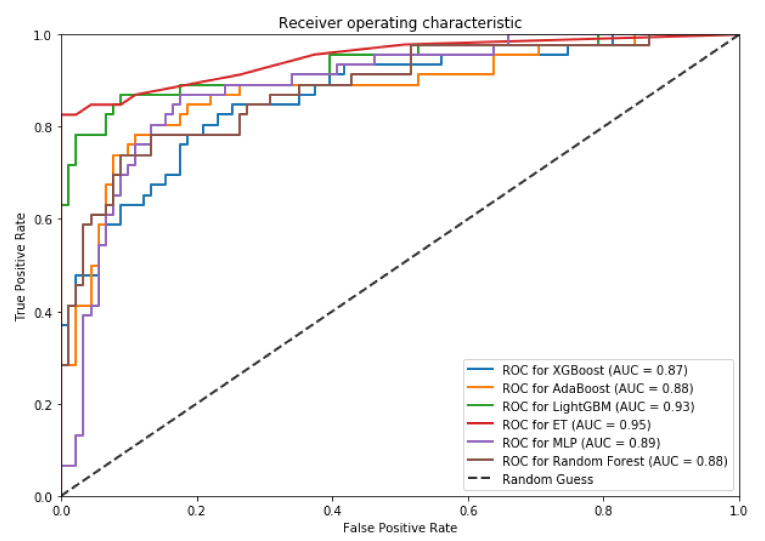
Receiver operator characteristic (ROC) curves using the independent test. The area under the curve (AUC) for each algorithm is indicated in parentheses.

We also plot the Receiver Operating Characteristic (ROC) to evaluate the output quality of the BiPepGlut both for 10-fold cross-validation and an independent test set. These plots are shown in Figure 4 and Figure 5, respectively.

These curves denote the *X*-axis as a false positive rate and *Y*-axis as a true positive rate. As shown in Figure 4 and Figure 5, ET performs consistently better than other classifiers. As shown in this figure, ET achieves the area under the curve (AUC) of 0.85 on the 10-fold cross-validation and AUC of 0.95 for the independent test set. Such results demonstrate the effectiveness of BiPepGlut using ET compared to other classifiers.

### 3.2. Comparison with State-of-the-Art Models

We compared our method with existing predictors that obtain the best results for the Glutarylation site prediction problem. To the best of our knowledge, we have identified three Glutarylation site predictors with the most promising results. In 2018, GlutPred [22] was developed using multiple feature extraction techniques along with maximum relevance and minimum redundancy feature selections to predict the Glutarylation sites. In the same year, iGlu-Lys [23] was developed using the finest features to predict the Glutarylation sites from the four-encodings method. Later on, RF-GlutarySite [25] was developed using sequence-based and physicochemical features and RF classifiers to predict the Glutarylation sites.

These three predictors are considered as the recent and most accurate predictors for the Glutarylation site prediction problem. To reproduce their results for our benchmark, we uploaded our sequences into their predictors and retrieved the performance of the predictors. Among these predictors, some are using 10-fold cross-validations, and others are using 6-fold, 8-fold, and 10-fold cross-validations during training. Consequently, their results may be exaggerated in independent test sets filtered from the entire data. Reproducing their results, we observed that the outcomes of those studies on independent test sets are better than assumed. Notwithstanding this, BiPepGlut was able to exceed even those obtained results.

We compare our method with these predictors (GlutPred, iGlu-Lys, and RF-GlutarySite). The results are shown in Table 3. As shown in this table, BiPepGlut achieves better results in terms of MCC, and the F1-score on the training set. The MCC and F1-score exceed 0.13 over previous predictor iGlu-Lys and 0.06 compared to RF-GlutarySite. The results of this comparison for the independent test set is shown in Table 4.

As shown in Table 4, BiPepGlut consistently performed better than other models investigated in this study. Our results demonstrate that the BiPepGlut achieves over 3% better ACC compared to these studies. Sensitivity and the F1-score improved by 11.8% and 0.16, compared to RF-GlutarySite [25]. As shown in this table, BiPepGlut comes with prominent outcomes in all matrices and performs better than those methods found in the literature. The significant improvement in sensitivity for our model demonstrates that BiPepGlut is able to identify Glutarylation sites by much more than those reported in previous studies. Given the performance of Glutarylation sites prediction, BiPepGlut can be considered as the most successful model compared to other studies found in the literature.

**Figure 6:**
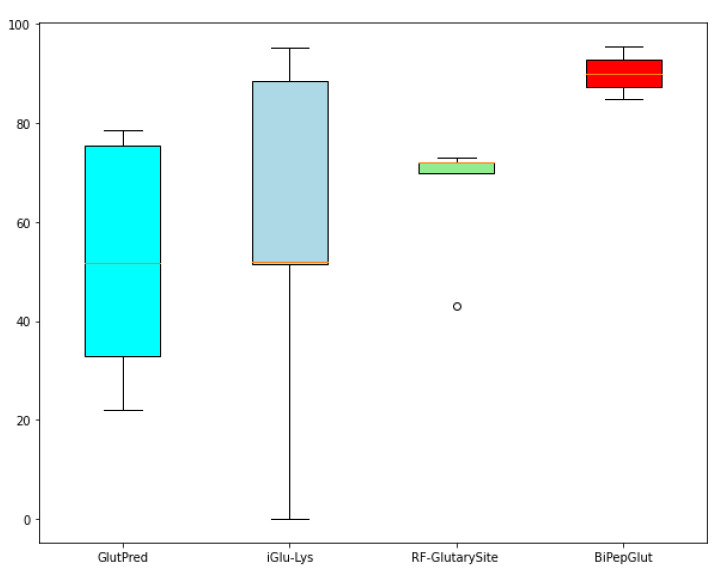
Comparing the results achieved using barplot among our model, BiPepGlut, GlutPred [22], iGlu-Lys [23], and RF-GlutarySite [25].

We also illustrate the barplot in Figure 6, which highlights the difference between the performance of BiPepGlut compared to GlutPred [22], iGlu-Lys [23], and RF-GlutarySite [25] in terms of accuracy.

Results illustrated in this figure demonstrate the effectiveness and accuracy of BiPepGlut in predicting Glutarylation sites compared to those methods found in the literature. BiPepGlut is implemented as an online predictor and is publicly available at: www.brl.uiu.ac.bd/bioglutarylation/. In addition, the data, code, and all the Supplementary Materials used to build BiPepGlut metis are publicly available at: https://github.com/Wakiloo7/BipepGlut.

### 3.3. Web Server Implementation

**Figure 7:**
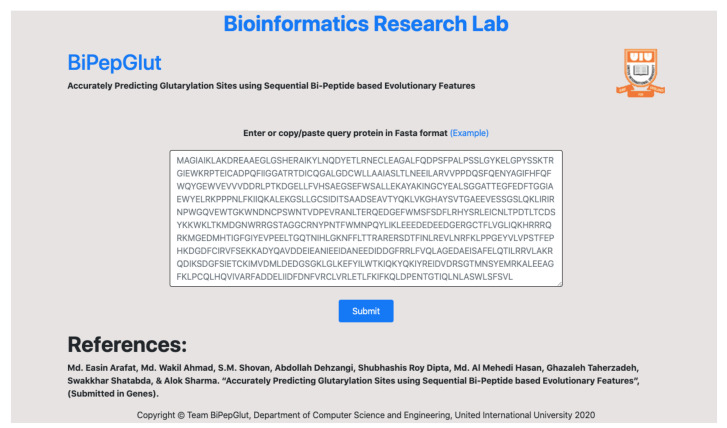
Screen-shot of BiPepGlut homepage.

We implemented BiPepGlut as a user-friendly and easy-to-use webserver. BiPepGlut is publicly available to use at: www.brl.uiu.ac.bd/bioglutarylation/. To use this predictor, the user has to provide a peptide sequence in fasta (.fsa) format. After uploading the sequence in BiPepGlut, PSSM files are generated from the server by using simultaneous iterations of PSI-BLAST where features are extracted and trained using the benchmark dataset. The goal of this predictor is to facilitate Glutarylation prediction. Figure 7 present the screen-shot of our online predictor.

## 4. Conclusions and Future Direction

In this study, we proposed a new method called BiPepGlut to predict the Glutarylation sites. To build BiPepGlut, we used bi-peptide-based evolutionary feature representation. We also used the Extra Tree classifier to build this model. Our results demonstrate that BiPepGlut can accurately predict the Glutarylation sites from Non-Glutarylation sites and improve the prediction results.

In the future, we aim to explore a wider range of features and include structural-based features to tackle this problem [39,54]. Such features are shown to be effective in solving similar problems in different studies. We also aim at comparing our extracted features with a wider range of feature extraction methods such as those extracted using iFeature [55] or BioSeq-Analysis [56,57]. In addition, we aim to find larger benchmarks that can allow us to use more advanced and complicated classifiers, such as Deep Learning, Convolutional Neural Network (such as DeepInsight [58]), and Recurrent Neural Network to enhance prediction accuracy even further. It is important to highlight that employing a larger benchmark will also enable us to provide more general and consistent results. Our future direction is to employ a larger benchmark as soon as it becomes available to further investigate the generality of our model. BiPepGlut is implemented as an online predictor and is publicly available at: www.brl.uiu.ac.bd/bioglutarylation/. In addition, the data, code, and all the Supplementary Materials used to build BiPepGlut metis are publicly available at: https://github.com/Wakiloo7/BipepGlut.

## Figures and Tables

**Table 1:** Name of measuring matrices used for comparing performances based on a 10-fold cross-validation.

*Model*	*ACC (%)*	*SN (%)*	*SP (%)*	*MCC*	*F1-Score*
*RF*	80.2%	63.4%	96.9%	0.64	0.76
*XGBoost*	79.7%	67.8%	91.5%	0.61	0.76
*LightGBM*	82.9%	74.2%	91.5%	0.67	0.81
*AdaBoost*	79.2%	74.7%	83.8%	0.59	0.78
*ET classifier*	81.5%	70.0%	92.9%	0.64	0.79
*MLP*	78.7%	75.4%	82.0%	0.58	0.78

**Table 2:** Name of measuring matrices used for comparing performances based on the independent-test set.

*Classifier Model*	*ACC (%)*	*SN (%)*	*SP (%)*	*MCC*	*F1-Score*
*RF*	79.6%	41.3%	98.9%	0.54	0.58
*XGBoost*	80.3%	45.7%	97.8%	0.55	0.61
*LightGBM*	91.2%	78.3%	97.8%	0.80	0.86
*AdaBoost*	85.4%	76.1%	90.1%	0.67	0.78
*ET Classifier*	92.0%	84.8%	95.6%	0.82	0.88
*MLP*	84.7%	76.1%	88.0%	0.64	0.76

**Table 3:** Comparison of the performance of BiPepGlut to existing Glutarylation predictor using 10-fold cross-validation.

Predictor Tool	ACC (%)	SN (%)	SP (%)	MCC	F1-Score
*GlutPred* [22]	74.9%	64.8%	76.6%	0.32	0.43
*iGlu-Lys* [23]	**88.4%**	50.4%	**95.2%**	0.51	-
*RF-GlutarySite* [25]	75.0%	**81.0%**	68.0%	0.50	0.73
*BiPepGlut*	81.5%	70.0%	92.9%	**0.64**	**0.79**

**Table 4:** Comparison of the performance of BiPepGlut to an existing Glutarylation predictor using the independent-test set.

Predictor Tool	ACC (%)	SN (%)	SP (%)	MCC	F1-Score
*GlutPred* [22]	75.4%	51.8%	78.5%	0.22	0.33
*iGlu-Lys* [23]	88.5%	51.4%	95.3%	0.52	-
*RF-GlutarySite* [25]	72.0%	73.0%	70.0%	0.43	0.72
*BiPepGlut*	**92.0%**	**84.8%**	**95.6%**	**0.82**	**0.88**
